# Guidelines for correlation coefficient threshold settings in metabolite correlation networks exemplified on a potato association panel

**DOI:** 10.1186/s12859-021-03994-z

**Published:** 2021-03-10

**Authors:** David Toubiana, Helena Maruenda

**Affiliations:** Departamento de Ciencias – Química, Centro de Espectroscopia de Resonancia Magnética Nuclear (CERMN), Pontificia Universidad Católica del Perú, Av. Universitaria 1801, Lima 32, Lima, Peru

**Keywords:** Metabolite correlation network, Threshold settings, Correlation coefficient, Pearson correlation, Potato association panel, Metabolism, Mouse heart metabolism

## Abstract

**Background:**

Correlation network analysis has become an integral tool to study metabolite datasets. Networks are constructed by omitting correlations between metabolites based on two thresholds—namely the *r* and the associated *p*-values. While *p*-value threshold settings follow the rules of multiple hypotheses testing correction, guidelines for *r*-value threshold settings have not been defined.

**Results:**

Here, we introduce a method that allows determining the *r*-value threshold based on an iterative approach, where different networks are constructed and their network topology is monitored. Once the network topology changes significantly, the threshold is set to the corresponding correlation coefficient value. The approach was exemplified on: (i) a metabolite and morphological trait dataset from a potato association panel, which was grown under normal irrigation and water recovery conditions; and validated (ii) on a metabolite dataset of hearts of fed and fasted mice. For the potato normal irrigation correlation network a threshold of *Pearson’s* |*r*|≥ 0.23 was suggested, while for the water recovery correlation network a threshold of *Pearson’s* |*r*|≥ 0.41 was estimated. For both mice networks the threshold was calculated with *Pearson’s* |*r*|≥ 0.84.

**Conclusions:**

Our analysis corrected the previously stated *Pearson’s* correlation coefficient threshold from 0.4 to 0.41 in the water recovery network and from 0.4 to 0.23 for the normal irrigation network. Furthermore, the proposed method suggested a correlation threshold of 0.84 for both mice networks rather than a threshold of 0.7 as applied earlier. We demonstrate that the proposed approach is a valuable tool for constructing biological meaningful networks.

## Background

Correlation-based network analysis (CNA) has become an integral tool for studying the coordinated behavior of metabolite profiles in plant sciences. Metabolite correlation networks (CN) are constructed by correlating each two pairs of metabolites across a set of different conditions or by exploiting the natural variability of mapping populations or collection of varieties, as they provide large sample size stabilizing the correlation and reducing the error rate, e.g. in a tomato introgression line mapping population [1, 2], a variety collection of sparkling wines [3], a diverse collection of *Arabidopsis* accessions [4], and a maize association panel [5]. The coordinated behavior of metabolites across diverse varieties gives insights into their genetic communalities. Each pairwise correlation is represented by a correlation-coefficient *r* ranging from -1 to 1*.* In addition, the significance of each correlation is evaluated by computing a *p*-value ranging from 0 to 1. In a metabolite CN, nodes represent metabolites and the edges between them represent the estimated correlation coefficients.

To construct metabolite CNs, threshold restrictions are applied to the correlation coefficients and their associated *p*-values to identify spurious correlations between metabolites. Subsequently, the non-significant correlations or edges, respectively, are removed from the network. Threshold settings for the associated *p*-values follow the rules of multiple hypotheses testing, such as a false discovery rate (FDR) [6]. However, guidelines for threshold settings of the correlation coefficient have not been well defined yet.

Ideally, edges between nodes in a CN reflect metabolic fluxes through a metabolic pathway. However, the relationship between metabolic pathways and the correlations between metabolites is not straight forward. Factors, such as short-term fluctuations caused by plant variability or internal noise may render into weak correlations. Systematic changes of the steady-state as well as aspects of cellular organization also need to be taken into account. Furthermore, the involvement of metabolites in multiple pathways and their extensive crosstalk within, makes it difficult to clearly affiliate metabolites to metabolic pathways in CNs. Thus, the correlation coefficient threshold cannot be universally set and must be instead adjusted to the system of study in order to extract meaningful biological data [7]. As a result, different studies have applied different selective threshold settings, e.g. Hu et al*.* constructed metabolite CNs for Osteoarthritis and control patients to identify significantly changing correlations between networks [8]. There the authors set a threshold for edges based on the *p*-value only. Via topological analysis of the difference network, they managed to highlight key metabolites that played an important role in governing the connectivity and information flow of the network. In [9] the authors used a moderate correlation coefficient threshold of 0.6 which enabled them to identify genes affecting free amino acids. In [10] a threshold of 0.7 was applied to highlight the differences between metabolite networks of fed and fasted mice. Yet again in [11] a rigorous correlation coefficient threshold of 0.8 was employed identifiying metabolic patterns for freezing tolerance in two *Brachypodium Sylvaticum* ecotypes. The selection of the correlation coefficient threshold, which allows meaningful biological interpretation, depends on the network topology rather than on the strength of the correlation coefficient itself. That being said, network properties associated with node connectivity alter (or better said stop altering) once a certain threshold has been reached. In other words, the selection of the correlation coefficient threshold depends on the distribution of the network’s numbers of edges at varying *p-*values, similar to the idea of choosing a *p*-value threshold based on the *p*-value distribution in the Benjamini–Hochberg FDR multiple hypotheses testing correction [6].

We have recently profiled the tuber of a potato association panel that was grown under normal irrigation (NI) and recovery (REC) treatments for 42 metabolites and also measured a set of 45 morphological traits of the plant. [12]. For each treatment, one CN was constructed, where the correlation coefficient threshold for both CNs was set to *Pearson’s* |*r*|≥ 0.4. In addition, the profiles were utilized for a genome wide association study (GWAS). Via the application of set theory to networks, a difference network was constructed, highlighting the difference set of the REC network over the NI network (REC ⊄ NI). In this perspective, the correlation between the metabolite fumaric acid and the morphological trait plant vigor was shown to be specific to the REC network. Next, we analyzed the single nuclear polymorphisms associated with fumaric acid and identified a gene coding for a RING domain protein on chromosome 1 in the potato genome and a gene coding for a zinc finger protein ZAT2 on chromosome 4. It was demonstrated before that both genes are essential for plants to cope with abiotic stresses [13–18].

In the current study, we demonstrate that the estimated correlation coefficient threshold of *Pearson’s* |*r*|≥ 0.4 was crucial for the establishment of a connection between fumaric acid and plant vigor, and by that for the identification of the aforementioned regulating genes. By in silico manipulations of the tuber CN and monitoring its connectivity between nodes, we define guidelines onto how to identify the proper correlation coefficient threshold for metabolite CNs. Last, we apply the proposed method on a mouse metabolite dataset to prove its efficacy on a dataset of different origin.

## Results

### Initial networks

We defined the NI and REC-CNs as weighted networks ${G}_{i}=({V}_{i},{E}_{i},w)$, where ${V}_{i}$ was the set of nodes corresponding to metabolites and morphological traits found in the dataset of treatment $i$, $E$ was the set of edges between them, and edge weights ($w:E\to R$) corresponded to the *Pearson* correlation coefficient. Thresholds for both networks were set to *Pearson’s* |*r*|≥ 0.4 and a *q*-value ≤ 0.05, removing spurious correlations. At these settings, the NI-CN had ${|V}_{NI}|=88$ nodes and $\left|{E}_{NI}\right|=438$ edges connecting them; the REC-CN was composed of $\left|{V}_{REC}\right|=90$ nodes and $\left|{E}_{REC}\right|=370$ edges. The connection between fumaric acid and plant vigor was present in the REC-CN but not in the NI-CN, as the corresponding correlation coefficients were computed with 0.458 and 0.013, respectively.

### Determining the correlation coefficient by testing the number of edges

As described above, the integration of a correlation into a CN depends on two threshold settings, namely the correlation coefficient and the associated *p*-value. In this and in other studies it was observed that the number of edges in a CN remains stable despite gradually increasing the *p*-value stringency settings until a certain correlation coefficient has been reached. In other words, the proposed method quantifies the number of edges that would be integrated into the CN dependent on the absolute correlation coefficient and its associated *p*-value. It does so in descending order at decrements of 0.1 of the correlation coefficient and ranging *p*-values of 0.05 to 0.01. Once the number of edges starts dropping at a certain correlation coefficient, the threshold is set to this value. We tested for significant changes in edge number by estimating the confidence intervals (CI) at 95% employing the modified Cox method [19] assuming non-normal distributions, such that:
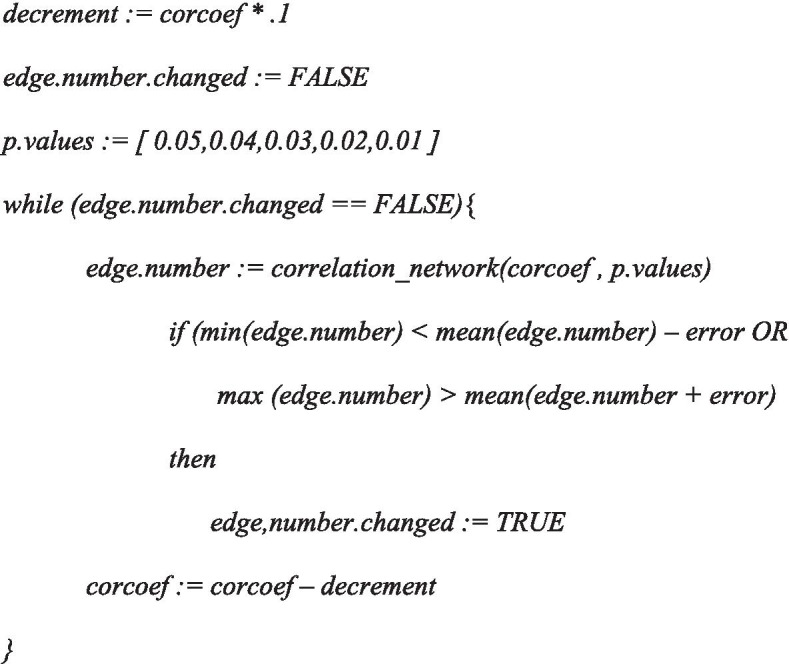


where the term *corcoef* corresponds to the initial correlation coefficient, *edge.number.changed* is a Boolean variable, *edge.number* is an array variable of function *correlation_network*, computing the number of edges of different CNs at parameters *corcoef* and *p.values,* and the *error* term is defined as CI / 2.

**Fig. 1:**
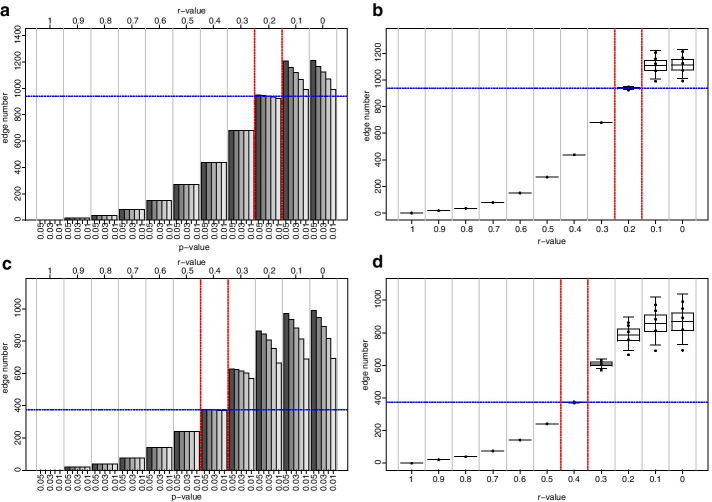
Edge number distribution of NI and REC networks, *r* = 1 to 0. Graphs on the left-hand side of the figure illustrate histograms of edge number in the NI and REC-CNs at different *r*-value to *p*-value combinations, at *r* = 1 to 0 at decrements of 0.1. Graphs on the right-hand side of the figure depict corresponding boxplots, where the centerlines represent the mean; box limits represent upper and lower standard error; whiskers represent 95% confidence intervals calculated by the modified Cox test. Grey vertical lines separate correlation coefficients, red dashed vertical lines represent proposed correlation coefficient threshold interval, blue horizontal lines represent the mean edge number at which threshold is set; **a** NI-CNs edge number histogram, **b** NI-CNs edge number boxplot, **c** REC-CN edge number histogram, **d** REC-CN edge number boxplot

To demonstrate this approach, we constructed a series of NI and REC networks at varying correlation coefficient and *p*-value settings and quantified the number of edges present (Fig. 1). The figure shows that the number of edges remained unchanged at varying *p*-values until the *Pearson’s* correlation coefficient dropped to 0.2 in the NI-CN (Fig. 1a and b), suggesting that the correlation coefficient threshold ought to be located somewhere in-between 0.2 and 0.3. At a correlation coefficient of 0.2 the edge number ranged between 923 and 948, the mean was estimated with 938.8, the upper limit of the 95% CI was calculated with 951.34, the lower limit with 926.45. For the REC-CN a significant drop was registered at a correlation coefficient of 0.4, suggesting that the threshold was supposed to be located somewhere in-between 0.4 and 0.5 (Fig. 1c and d). The associated values were estimated with mean = 373.4, the upper limit of the CI = 376.66, the lower limit of the CI = 370.18, the minimum = 370, and the maximum = 376.

### Fine-tuning computes the correlation coefficient thresholds with 0.23 and 0.41

**Fig. 2:**
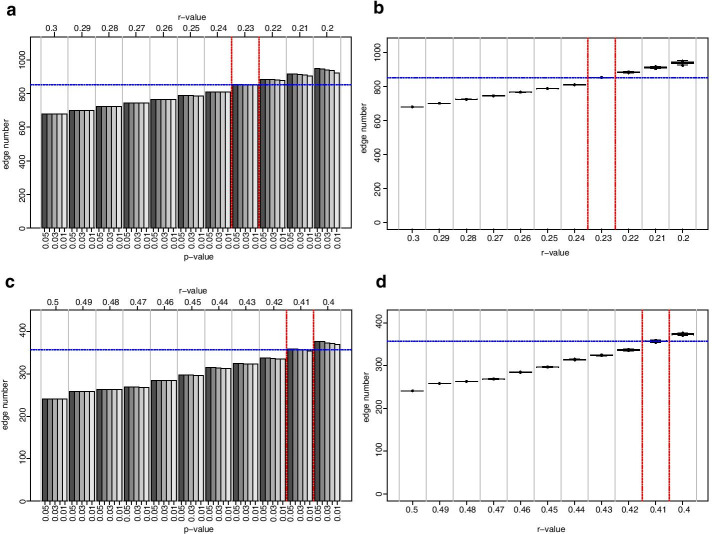
Edge number distribution of NI and REC networks, *r* = 0.3 to 0.2 and 0.5 to 0.4. Graphs on the left-hand side of the figure illustrate histograms of edge number in the NI and REC-CNs at different *r*-value to *p*-value combinations, at *r* = 0.3 to 0.2 and 0.5 to 0.4 at decrements of 0.01, respectively. Graphs on the right-hand side of the figure depict corresponding boxplots, where the centerlines represent the mean; box limits represent upper and lower standard error; whiskers represent 95% confidence intervals calculated by the modified Cox test. Grey vertical lines separate correlation coefficients, red dashed vertical lines represent proposed correlation coefficient threshold interval, blue horizontal lines represent the mean edge number at which threshold is set; **a** NI-CNs edge number histogram, **b** NI-CNs edge number boxplot, **c** REC-CN edge number histogram, **d** REC-CN edge number boxplot

Next, we investigated CNs at different *r*-values in the range of 0.2 to 0.3 for the NI-CN and in the range of 0.4 to 0.5 for the REC-CN (Fig. 2). As before, the* r* threshold value was defined as the *r*-value when the minimum or maximum edge number was located outside the confines of the corresponding CI. This behavior occurred for the NI-CN at a correlation coefficient threshold *Pearson’s* |*r*|≥ 0.23. Here, the edge number ranged between 850 and 853, the mean was calculated with 851.8, the CI lower limit was computed with 850.18 and the CI upper limit with 853.42 (Fig. 2). For the REC-CN this behavior was observed at *Pearson’s* |*r*|≥ 0.41. At this *r*-value the edge number ranged between 354 and 359, the mean was computed with 357, the CI upper limit with 359.65 and the CI lower limit with 354.37. Consequently, the *r*-value threshold for the NI-CN was proposed with 0.23 and for the REC-CN with 0.41.

### Bootstrapping analysis confirms correlation coefficient thresholds

**Fig. 3:**
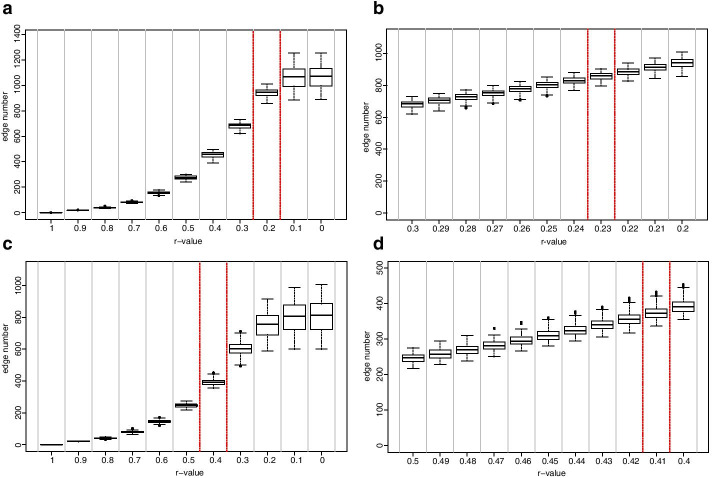
Edge number distribution of bootstrapped NI and REC networks, *r* = 1 to 0. **a** NI-CNs edge number boxplot from bootstrapping analysis at different *r*-values, at *r* = 1 to 0 at decrements of 0.1. **b** NI-CNs edge number boxplot from bootstrapping analysis at different *r*-values, at *r* = 0.3 to 0.2 at decrements of 0.01. **c** REC-CNs edge number boxplot from bootstrapping analysis at different *r*-values, at *r* = 1 to 0 at decrements of 0.1. **d** REC-CNs edge number boxplot from bootstrapping analysis at different *r*-values, at *r* = 0.5 to 0.4 at decrements of 0.01. In the boxplots, centerlines represent the median; box limits represent upper and lower quartiles; whiskers represent 1.5 × interquartile range. Bootstrapping was run 100 times with 80% of the samples allowing replacement. Grey vertical lines separate correlation coefficients, red dashed vertical lines represent proposed correlation coefficient threshold interval

To verify the proposed correlation coefficient threshold settings, we employed bootstrapping with replacement; such that 100 NI and REC-CNs were generated with 80% of the samples randomly selected, where one sample could be part of the sample subset more than once. Performing this analysis, we wanted to validate whether the estimated threshold was due to chance or indeed the result of the network topology at the proposed threshold even at a reduced set of samples (80%). As before, the analysis was divided into two cycles. For the first cycle the edge numbers of all networks were quantified at *r*-values from 1 to 0 at decrements of 0.1 with varying *p*-values (Fig. 3a and c). For the second cycle the edge numbers for the NI-CN were quantified at *r*-values ranging from 0.3 to 0.2 and for the REC-CN at at *r*-values ranging from 0.5 to 0.4 with varying *p*-values (Fig. 3b and d). For both latter analyses decrements of 0.01 were used. The boxplot of the NI-CNs illustrates an increased range of edge number the lower the *r*-value became, revealing an increased range from values 0.4 downwards (Fig. 3a). At an *r-*value of 0.3 the complete range for all 100 NI-CNs was calculated with 620 to 730 (range = 110) edges present in the networks. At an *r*-value of 0.2 the minimum edge number was estimated with 858, while the maximum edge number was 1,010, showing an increased range (152). At an *r*-value of 0.23 the complete range for all 100 NI-CNs was calculated with 797 to 905 (range 108) edges present in the networks (Fig. 3b). For the REC-CN an increased range was specifically visible after the correlation coefficient dropped beneath 0.5, i.e. at *Pearson’s r* = 0.5 the range of edge numbers in all 100 REC-CNs was 58 (min = 217, max = 275), while at *r* = 0.4 the range increased to 98 (min = 355, max = 453 – Fig. 3c). We further investigated *r*-values in-between 0.5 to 0.4 (Fig. 3d), demonstrating a steady increase of median edge numbers the lower the correlation coefficient became. At the targeted *r*-value of 0.41 the minimum edge numbers was estimated with 337 and the maximum edge number with 421 (range 84). Furthermore, the boxplot revealed increased CIs, indicative for greater standard errors attributed to the increased edge numbers at lower correlation coefficients. These findings underscored the original presupposition of a correlation coefficient threshold setting of *Pearson’s* |*r*|≥ 0.23 in the NI-CN and *Pearson’s* |*r*|≥ 0.41 in the REC-CN.

**Fig. 4:**
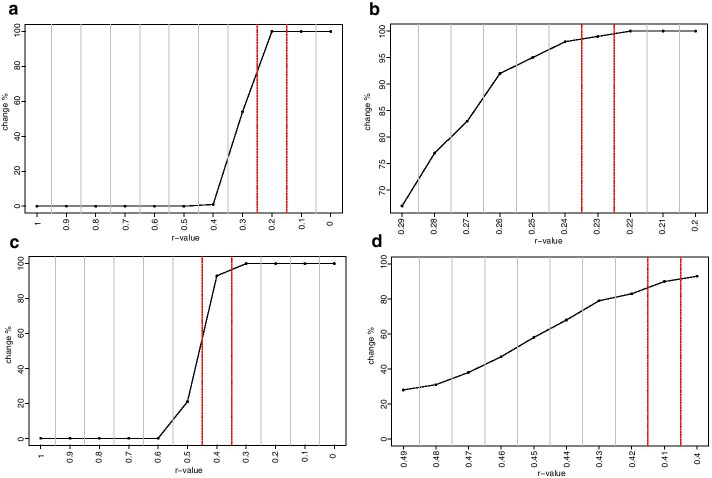
Relative number of significantly changing bootstrapped NI and REC-CNs. The figure illustrates the relative number of significantly changing edge numbers in bootstrapped CNs. **a** NI-CNs relative change in edge number based on bootstrapping analysis at different *r*-values, at *r* = 1 to 0 at decrements of 0.1. **b** NI- relative change in edge number based on bootstrapping analysis bootstrapping analysis at different *r*-values, at *r* = 0.3 to 0.2 at decrements of 0.01. **c** REC-CNs relative change in edge number based on bootstrapping analysis at different *r*-values, at *r* = 1 to 0 at decrements of 0.1 **d** REC-CNs relative change in edge number based on bootstrapping analysis at different *r*-values, at *r* = 0.5 to 0.4 at decrements of 0.01. The confidence interval was estimated by the modified Cox test at different *r*-value to *p*-value combinations (see main text for details). Bootstrapping was run 100 times with 80% of the samples allowing replacement. Grey vertical lines separate correlation coefficients, red dashed vertical lines represent proposed correlation coefficient threshold interval. **a** Bootstrapped NI-CNs at *r* = 1 to 0 at decrements of 0.1; **b** Bootstrapped REC-CNs at *r* = 1 to 0 at decrements of 0.1; **c** Bootstrapped REC-CNs at *r* = 0.5 to 0.4 at decrements of 0.01

**Fig. 5:**
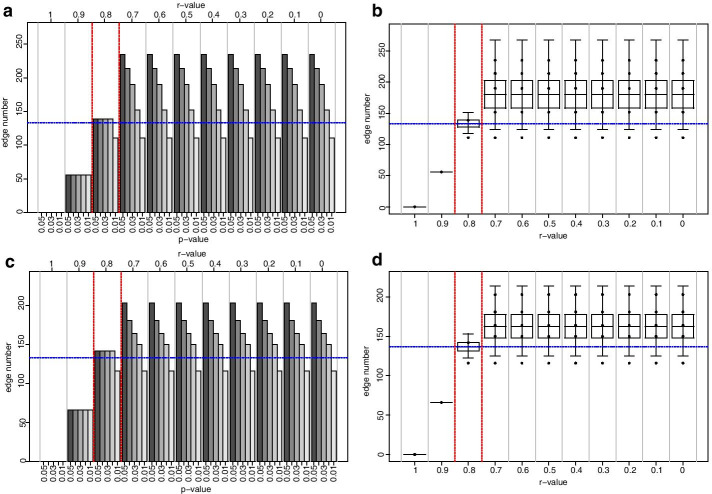
Edge number distribution of Mouse_fed_ and Mouse_fasted_ networks, *r* = 1 to 0. Graphs on the left-hand side of the figure illustrate histograms of edge number in the of Mouse_fed_ and Mouse_fasted_ at different *r*-value to *p*-value combinations, at *r* = 1 to 0 at decrements of 0.1. Graphs on the right-hand side of the figure depict corresponding boxplots, where the centerlines represent the mean; box limits represent upper and lower standard error; whiskers represent 95% confidence intervals calculated by the modified Cox test. Grey vertical lines separate correlation coefficients, red dashed vertical lines represent proposed correlation coefficient threshold interval, blue horizontal lines represent the mean edge number at which threshold is set; **a** Mouse_fed_-CNs edge number histogram, **b** Mouse_fed_-CNs edge number boxplot, **c** Mouse_fasted_-CN edge number histogram, **d** Mouse_fasted_-CN edge number boxplot

Next, we used the bootstrapped CNs and computed the CI for each correlation coefficient at varying *p*-values employing the modified Cox test (Fig. 4). For the first cycle, 1% (empirical *p* = 0.99) of all NI-CNs at an *r*-value of 0.4 (Fig. 5a) revealed minimum or maximum edge numbers beyond their estimated CI (Fig. 4a); at *r* = 0.3, the number rose to 54%, equivalent to an empirical *p*-value of 0.46. At *r* = 0.2, all networks showed to have minimum or maximum edge numbers beyond their estimated CI (empirical *p* < 0.01). During the second cycle we inspected in particular the targeted *r*-value of 0.23, revealing that 98% (empirical p-value 0.02) of all networks had minimum or maximum edge numbers beyond their estimated CI (Fig. 4b) For the bootstrapped REC-CNs, significant changing edge numbers as suggested by the CI started to occur at *r* = 0.5 (Fig. 4c). Here, 21% of the CNs showed significant changes (empirical *p* = 0.79); at *r* = 0.4, 93% of all REC-CNs exerted significant changes (empirical *p* = 0.07), and at *r* = 0.3, all networks (empirical *p* < 0.01) had significantly changing edge numbers. For the second cycle we investigated the number of bootstrapped CNs associated with the proposed cutoff *r*-value of 0.41. At this value, 90% of all networks revealed to have significantly changing edge numbers, which equated to an empirical *p*-value of 0.1. By setting the *p*-value cutoff threshold to ≤ 0.1 the bootstrapping analysis confirmed the proposed NI-CN and REC-CN correlation coefficient threshold.

### Method validation on mouse metabolite dataset

**Fig. 6:**
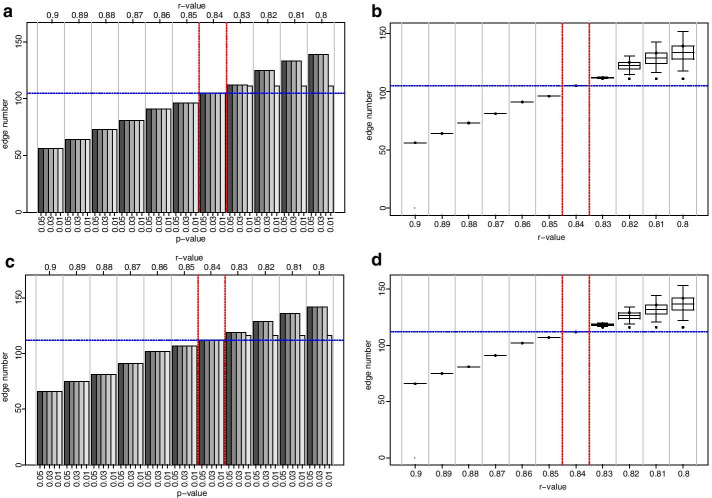
Edge number distribution of Mouse_fed_ and Mouse_fasted_ networks, *r* = 0.9 to 0.8. Graphs on the left-hand side of the figure illustrate histograms of edge number in the Mouse_fed_ and Mouse_fasted_ at different *r*-value to *p*-value combinations, at *r* = 0.9 to 0.8 at decrements of 0.01. Graphs on the right-hand side of the figure depict corresponding boxplots, where the centerlines represent the mean; box limits represent upper and lower standard error; whiskers represent 95% confidence intervals calculated by the modified Cox test. Grey vertical lines separate correlation coefficients, red dashed vertical lines represent proposed correlation coefficient threshold interval, blue horizontal lines represent the mean edge number at which threshold is set; **a** Mouse_fed_-CNs edge number histogram, **b** Mouse_fed_-CNs edge number boxplot, **c** Mouse_fasted-_CN edge number histogram, **d** Mouse_fasted_-CN edge number boxplot

To validate the proposed method on a dataset of different biological background, we utilized the mouse metabolite datasets from Batushansky et al. [10]. There the authors tested the effect of fasting in the hearts of mice. For one of the analyses used in the study, the authors constructed CNs for two conditions, i.e. fed and fasted mice. To highlight the differences between networks intersection edges were identified. Networks were constructed at an absolute *r*-value threshold of ≥ 0.7. Here, we used the datasets for fed and fasted mice and ran it through our correlation-coefficient threshold pipeline. For the first cycle, our method suggested that the correlation coefficient threshold ought to be located in between *r*-values 0.8 and 0.9 for both networks (Fig. 5). For the second cycle, the *r*-value for which the edge number was located within the lower and upper CI was determined with 0.84 for both networks (Fig. 6). At these settings, the Mouse_*Fed*_-CN had ${|V}_{Fed}|=42$ nodes and $\left|{E}_{Fed}\right|=105$ edges connecting them; the Mouse_*Fasted*_-CN was composed of $\left|{V}_{Fasted}\right|=42$ nodes and $\left|{E}_{Fasted}\right|=112$ edges. In the original study the authors identified eight edges intersecting both networks. Here, we identified 17 intersecting edges, containing all edges of the original study (Additional file 1: Supplementary Data S1).

## Discussion

The construction of metabolite CNs is a non-trivial undertaking. In contrast to weighted gene co-expression networks [20], where all edges are kept within the network, the aim of metabolite CNs is to eliminate some of the correlations [21]. As such, if the correlation coefficient threshold is set too high, valuable biological data may be lost, while if the correlation coefficient threshold is set too low the plethora of edges may have a confounding effect, rendering it difficult to identify the most viable biological information associated with the data at hand. It is therefore highly important to set the correlation coefficient threshold appropriately so that meaningful biological conclusions can be derived. However, the correlation coefficient threshold for metabolite CNs cannot be universally set. Instead, it must be determined dynamically in accordance to the studied system. Although different studies have already applied different correlation coefficient thresholds to construct metabolite CNs, e.g. [1, 4, 8–11, 22–24], a set of rules on how to determine them has not been established, yet. Here we introduced an approach that allows researchers to select a correlation coefficient threshold suitable to their studied system.

In our recent study on a potato association panel we constructed a CN on tuber metabolites and plant morphological traits, where we applied a correlation coefficient threshold of 0.4 [12]. At this setting we identified a critical connection between fumaric acid and plant vigor, which enabled us to identify essential genes aiding the plant to cope with abiotic stresses. Using the same potato dataset, in the current study, we demonstrated how to select the adequate correlation coefficient threshold based on an iterative approach, during which the network topology and specifically its associated edge number was monitored. A series of CNs were constructed, where different absolute correlation coefficients (from 0 to 1) were combined with a set of different *p*-values. To determine the *r*-value threshold a significant change had to be detected. We defined a significant change as the excess of the range of edge numbers beyond the confines of the corresponding CI of a CN. Once this criterion was fulfilled, the *r*-value threshold was set.

The initial analysis with increments of 0.1 between *r*-values from 0 to 1 suggested a threshold 0.2 for the NI-CN and a threshold 0.4 for the REC-CN (Fig. 1). The second cycle of our proposed method suggested an *r*-value threshold of 0.23 for the NI-CN and 0.41 for the REC-CN (Figs. 1 and 2). Bootstrapping analysis with 100 CNs based on 80% of the samples confirmed the proposed thresholds (Figs. 3 and 4). At these threshold settings the crucial connection between fumaric acid and plant vigor was still present in the REC-CN but not in the NI-CN as suggested in [12]. To validate the proposed method, it was also applied to heart metabolite datasets of fed and fasted mice [10]. The original study proposed a threshold of 0.7 for both CNs. To compare the CNs a network intersection was generated. Our analysis suggested a threshold 0.84 for both CNs. Although our proposed threshold was significantly higher than in the original study, we showed a similar edge intersection.

The proposed thresholds for the four different CNs stretched over a wide range of *r*-values, starting at a correlation coefficient as low as 0.23 for the NI-CN and reaching a correlation coefficient as high as 0.84 for the two mouse CNs. To identify a property that could potentially be key to this finding, we computed a number of network properties for each CN. Network properties derived from node degree (e.g. average degree, edge to node ratio, density, clustering coefficient) varied as much as the proposed correlation coefficient thresholds themselves. However, two other network properties provided interesting results that could potentially be the key elements for correlation coefficient threshold detection, namely: i) the network diameter, which is the maximum geodesic distance between any pair of nodes in a graph; and ii) the network assortativity coefficient, which is the correlation coefficient of degree between pairs of connected nodes [25]. It tells in a concise fashion how nodes are preferentially connected to each other. The diameter for the NI-CN was computed with 6 and its assortativity coefficient with 0.45, for the REC-CN the diameter was 9 and its assortativity coefficient = 0.36, for the Mouse_*Fed*_-CN the diameter was 9 and its assortativity coefficient = 0.47, and for the Mouse_*Fasted*_-CN the diameter was calculated with 8 and its assortativity coefficient with 0.52. Despite the different topologies of the four CNs these two network properties revealed comparable values. We believe that this finding should be further investigated.

## Conclusions

We demonstrated that the approach developed in this study is a valuable tool for the determination of the correlation coefficient threshold for the construction of metabolite CNs. We applied our method to metabolite datasets of different biological background and the thresholds suggested varied from 0.23 over 0.41 to 0.84. Although the newly proposed *r-*values differed from the values utilized in the original studies, it still allowed us to obtain the same biological conclusions. It is therefore that the network topology of CN determines the biological interpretation, rather than the strength of the correlation coefficient itself. For this reason, we suggest treating CNs as unweighted graphs once the correlation coefficient has been established and non-significant correlations have been removed.

## Methods

### Datasets acquisition and processing

Datasets for metabolites and morphological traits were adopted from [12]. Preprocessing and quantification of metabolites and morphological traits were performed as described therein.

### CN settings

The generation of the network was based on the correlation analysis of all metabolites and morphological traits. The Pearson correlation was chosen to estimate correlation coefficients. To construct the initial NI and REC networks, the correlation coefficient threshold was set to 0.4 as previously described. The corresponding *p*-value 0.05 was adjusted via multiple hypotheses testing correction. The mice CNs were constructed as described in [10].

### Confidence Interval estimation

The estimation of the CI is based on a normal distribution. As the data in this study violated this assumption we employed the CI estimation based on the modified Cox method, which log-transforms the data prior to estimation [19]. It also applies *t*-values rather than *z*-values.

### Bootstrapping

To statistically verify the approach presented in the current study for correlation coefficient threshold settings, we employed bootstrapping with random sample replacement. Bootstrapping was performed 100 times with 80% of the samples available in the NI and REC datasets.

### Availability and requirements

- Project name: Correlation coefficient threshold determination in correlation-based networks
- Project home page: https://github.com/toubiana/correlation_coefficient_threshold
- Operating system(s): Platform independent
- Programming language: R
- Other requirements: The source code operates in R without any dependencies.
- License: ACADEMIC PUBLIC LICENSE
- Any restrictions to use by non-academics: e.g. licence needed

## Supplementary Information

**Additional file 1.** Network intersection. File description: Network intersections of mouse CNs as identified in the current study and in Batushansky et al. [10].

## Data Availability

All data and material associated with the current study can be found in the supplementary data of [12].

## Abbreviations

CI: confidence interval
CNA: correlation-based network analysis
CN: correlation networks
FDR: false discovery rate
GWAS: genome wide association study
NI: normal irrigation
REC: recovery
